# Predictors and Barriers to Full Vaccination among Children in Ethiopia

**DOI:** 10.3390/vaccines6020022

**Published:** 2018-04-10

**Authors:** Yemesrach A. Tefera, Abram L. Wagner, Eyoel B. Mekonen, Bradley F. Carlson, Matthew L. Boulton

**Affiliations:** 1 Department of Public Health, St. Paul’s Hospital Millennium Medical College, Addis Ababa 1271, Ethiopia; yemieye197@gmail.com (Y.A.T.); eyulovethiopia@gmail.com (E.B.M.); 2 Department of Epidemiology, School of Public Health, University of Michigan, 1415 Washington Heights, Ann Arbor, MI 48109, USA; bcarlson@umich.edu (B.F.C.); mboulton@umich.edu (M.L.B.); 3 Department of Internal Medicine, Division of Infectious Diseases, University of Michigan Medical School, 1500 East Medical Center Drive, Ann Arbor, MI 48109, USA

**Keywords:** Ethiopia, vaccination coverage, religion, vaccine hesitancy, perinatal care

## Abstract

Predictors of immunization status outside of large cities in Ethiopia are not well known, and Muslims have lower vaccination coverage. The aim of this study is to assess factors associated with full immunization among children 12–23 months in Worabe, Ethiopia, a Muslim-majority community. A cross-sectional study is conducted in summer 2016. Multivariable logistic regression was used to assess the significance of predictors of full immunization. Among 484 children, 61% are fully vaccinated. Children whose mothers had fewer antenatal care (ANC) visits have decreased odds of full vaccination (zero visits: odds ratio (OR) = 0.09; one visit: OR = 0.15; two visits: OR = 0.46; three visits: OR = 0.89). The most common reasons that the mother gave for not vaccinating the child are fear of side reactions (36%), being too busy (31%), or hearing rumors about vaccines (28%). Local interventions incorporating interventions with religious authorities could raise awareness in the community of the importance of childhood immunizations and ANC visits.

## 1. Introduction

Vaccinations are one of the most cost-effective and impactful health interventions used worldwide, and have resulted in dramatic declines and regional elimination of many serious childhood infectious diseases [1]. Of the nearly 135 million children born globally in 2015, 19 million children (14%) had not received a third dose of diphtheria–tetanus-pertussis vaccine (DTP), a sentinel vaccine indicative of immunization program performance. Insufficient coverage with DTP and other recommended vaccines results in 1.5 million childhood deaths throughout the world from vaccine-preventable diseases each year [2]. Due to its large birth cohort (over three million), and relatively low immunization coverage (86% for DTP3) [3], Ethiopia counts among the top ten countries with the greatest number of children who lack DTP3 vaccination [4]. A model of mortality in children <5 years of age in Ethiopia in 2015 estimated that 13.69% of deaths were attributable to lower respiratory infections, 7.73% to diarrhea, 4.15% to meningitis, 3.76% to whooping cough, and 3.74% to measles [5,6].

Nonetheless, Ethiopia is notable for rapidly improving and developing its immunization programs in recent years. According to estimates from the World Health Organization (WHO) and the United Nations’ Children’s Fund (UNICEF), coverage of DTP3 in Ethiopia increased from 61% in 2011 to 86% in 2015 [3], and the government has recently added more recently developed vaccines, such as the pneumococcal conjugate (PCV) and rotavirus vaccines, to the country’s publicly-funded Expanded Program on Immunization (EPI) [7]. Vaccines on Ethiopia’s EPI are provided free of charge when administered in public health facilities, whereas service and vaccine fees are incurred at private health facilities located primarily in major urban centers. Within a study of vaccine hesitancy from 67 countries, participants from Ethiopia had some of the highest levels of trust in vaccinations, responding positively to questions like “vaccines are important for children to have”, and “overall I think vaccines are effective” [8]. However, vaccine hesitance has become relatively high in other low- and middle-income countries, and a better understanding of how vaccine hesitancy is developing in diverse contexts, and how it relates to traditional barriers to receipt of vaccines, is becoming increasingly important. 

Although overall vaccination coverage with the EPI vaccines has improved in Ethiopia, there are substantial regional disparities in vaccination coverage. For instance, coverage with the third dose of pentavalent vaccine ranges from 96.4% in Addis Ababa, the capital city and a large urban center, to 23.0% in Afar, a rural area [7]. Studies from several areas of Ethiopia have identified maternal education, urbanicity, maternal health care utilization, and mothers’ awareness of vaccination and perceived health care support as significant associations with immunization coverage [9,10,11]. Studies from other countries in Sub-Saharan Africa, India, and Bangladesh have also shown that utilization of maternal health care service including antenatal care (ANC) visits, residence in urban areas, low literacy rates, and high fertility rates are all associated with a child’s immunization status [12,13,14].

Ethiopia is home to a highly diverse populace, and the barriers to full vaccination and risk factors for under-immunization likely vary across regions and ethnic groups. Remote regions far from the capital, Addis Ababa, are especially uncharacterized with regard to predictors for vaccination, as are minority groups, such as Muslims, who are known to have lower rates of immunization [15]. Although on a national and global level, there are several major trends in childhood vaccination, such as that individuals with lower socioeconomic status encounter greater barriers to attending a vaccine clinic [16,17], there could be other influences identified within local populations, which tend to be more religiously and ethnically homogenous. Additionally, although vaccine hesitancy has largely been studied in Western countries, identifying its occurrence in low- and middle-income countries at an early stage could help mitigate long-term declines in confidence towards immunization programming in low resource settings [18]. This study undertakes a survey of mothers of young children in Worabe, which is a Muslim-majority town in the Southern Nations, Nationalities, and Peoples’ Region of Ethiopia. We estimate the proportion of children who are fully immunized, identify risk factors for being under immunized, and explore socioeconomic and knowledge-related barriers to full participation in the vaccination program. 

## 2. Materials and Methods

### 2.1. Study Population

This cross-sectional study took place during July and August 2016 in Worabe, a town located in the Southern Nations, Nationalities, and Peoples’ Region, which is one of nine regional states in Ethiopia located. The town comprises 27,852 residents according to 2007 Ethiopian census, of whom 15% are under five years of age. 

In Worabe are two *kebele*, small administrative units in Ethiopia analogous to a neighborhood or a ward. In each *kebele*, a random starting point was chosen using a random number chart, and households were enumerated from this random starting point to the limits of the *kebele*. Ten trained staff, who were female community health workers, enrolled participants and administered the in-person interviews. All interviewers attended a one-day training session, which included topics on questionnaire content, participation selection procedures, and research ethics. Interviewers began at the random starting point and checked if the household had a child between 12 and 23 months, which was the sole inclusion criteria of the study. In instances where two or more children between the ages of 12 and 23 months were present in the household, the youngest child within the allowable age range was selected. Thereafter, each adjacent household was visited until the sample size quota (270) for each *kebele* was met. The mother of the enrolled child was interviewed. 

A sample size of 541 children was targeted, based on an estimate of 80% full vaccination in the study, and a desired margin of error of 5% around a 95% confidence interval. We assumed a 10% non-response rate and a design effect of 2.

### 2.2. Questionnaire

The quantitative questionnaire was pre-tested in Worabe Hospital on 54 participants, and administered by Health Extension Workers. Interviewers conducted the structured questionnaire, initially developed in English and later translated into Amharic, for data collection. Questionnaire development was based on questions adapted from the Demographic and Health Survey (DHS) of Ethiopia, in combination with a previous survey of EPI coverage in selected Ethiopian locations that has been published elsewhere [19]. The questionnaire addressed multiple areas including sections on socioeconomic characteristics of mother and child; utilization of ANC and health institution delivery by mothers; child characteristics; mother’s knowledge of vaccination and vaccine preventable diseases; and immunization history of the child. Mothers whose children were missing at least one vaccination dose were asked why their child had missed this dose; they were given a list of reasons and could select multiple reasons. Data on immunization history were collected either from vaccination cards or maternal recall. 

For purposes of the questionnaire and the study, full immunization was defined as a child 12–23 months of age who had received one dose Bacillus Calmette-Guerin (BCG); three doses of a combination pentavalent vaccine, which includes DTP, hepatitis B, and *Haemophilus influenzae* type b; three doses of PCV; two doses rotavirus vaccine; three doses oral polio vaccine (OPV); and one dose measles vaccine [7]. 

### 2.3. Statistical Analysis

We describe the population by demographic characteristics, and the proportion fully vaccinated. Mothers’ reasons for not vaccinating child were tallied and grouped into three categories: lack of information, lack of motivation, and obstacles. In the multivariable logistic regression, we included demographic and economic characteristics, along with variables related to mothers’ knowledge of vaccines. Variables were included in the model based on a priori considerations. All questions listed in the survey were hypothesized to be related to vaccination, but we only included factors for which there were adequate numbers (i.e., at least 20) of children in each category to precisely estimate odds ratios (ORs) and 95% confidence intervals (CI) from the logistic regression. We excluded some variables from the multivariable analysis (parity, mother has younger children, and mother has older children) because of substantial overlap with another factor—birth order. Questions related to source of information about vaccines and knowledge about number of vaccine sessions and age when childhood immunization begin were additionally not included to present a parsimonious model. Significance testing was done at an α level of 0.05. Data were analyzed in SPSS version 20 (IBM, Armonk, NY, USA).

### 2.4. Ethical Approval

This survey was conducted in compliance with international norms in study ethics, such as the Declaration of Helsinki. The study proposal was approved by the Institutional Review Board of St Paul’s Hospital Millennium Medical College in Ethiopia (approval #P.M.23/17). Permission to undertake the study was obtained from Worabe Health Bureau. Before enrolling participants into the study, interviewers obtained verbal informed consent from mothers of the young children. The informed consent document included information on the survey content, confidentiality of data, and the voluntary nature of survey participation. 

## 3. Results

A total of 540 mothers of children aged 12–23 months were approached and 484 (90%) agreed to participate in the study. The mean age of the participating children was 17.4 months with standard deviation (SD) of 4.1 months. The socioeconomic characteristics of study participants are summarized in Table 1. Most children belonged to families with multiple children; 149 (31%) of the children were the first-born, but 150 (31%) were the fourth or greater child in the family. The mothers’ ages ranged from 18 to 47, but most were either 21–25 years of age (140, 29%) or 26–30 years of age (181, 37%). The degree of education in the study population varied; 36% (171) had no formal education, but 12% (59) had a college degree. About half (51%) of mothers were house wives, the rest were students (1%) or had jobs outside of the home. About one-third (162, 33%) owned farm land. Almost all (474, 98%) of the mothers were married, and 89% (430) were Muslim.

A plurality of mothers (208, 43%) had at least four ANC visits, but many only had one (104, 21%), or no (26, 5%) ANC visits. The distance between home and vaccination site was roughly divided into three groups: those for whom it took <30 min (157, 32%), 30–59 min (181, 37%), and an hour or more (146, 30%). A total of 297 (61%) of the study participants were fully vaccinated.

Full vaccination coverage noticeably varied across some of the demographic characteristics. House wives had children with higher coverage of full immunization status (63%) than certain other occupations such as merchants (51%) or public/private employees (56%). Families with farm land also had greater coverage of full immunization status (70%) than those without (57%). There was a strong gradient between ANC visits and vaccination status; only 15% of children whose mothers had had no ANC visits were fully vaccinated, compared to 77% of those whose mothers had at least four visits. Lastly, families whose home was at least an hour from the vaccination site were less likely to be fully vaccinated (56%) than families whose home was between 30 and 59 min away (67%); there was no significant difference between living 30–59 min away and <30 min away.

Table 2 shows information about the mothers’ health care utilization and knowledge of vaccines. Most mothers (94%) took their child to health facilities for regular check-ups and most (92%) would take their child to a health facility if he or she was sick. Mothers heard about vaccines from several sources including Health Extension Workers (61%), TV (26%) and community leaders (25%) (selections were not mutually exclusive). The content of that information about vaccines was related to the benefits of vaccination (81%), where to get vaccinated (61%), and age appropriateness for vaccination (42%). A total of 260 (54%) of mothers hesitated to vaccinate their child at some point; the most common reasons they gave were excessive waiting time (179, 37%), dislike of health professional(s) (153, 32%), and fear of needles (142, 29%).

In the multivariable logistic analysis (Table 3), four factors were significantly associated with full vaccination: mother’s occupation (*p* = 0.0325), owning farm land (*p* = 0.0008), number of ANC visits (*p* < 0.0001), and distance to vaccination site (*p* = 0.0440). Mothers who worked outside the home had 0.58 times the odds of having children fully vaccinated compared to housewives (95% CI: 0.35, 0.96), and families with farm land had 2.51 times greater odds of fully vaccinated children compared to families without (95% CI: 1.46, 4.30). As in the bivariate analysis, there was a dose–response relationship between number of ANC visits and vaccination status. Compared to children whose mothers had at least four ANC visits, the odds of full vaccination status were 0.89 times as high with three visits (95% CI: 0.48, 1.67), 0.46 times as high with two visits (95% CI: 0.23, 0.92), 0.15 times as high with one visit (95% CI: 0.08, 0.29), and only 0.09 times as high with zero visits (95% CI: 0.03, 0.32). Children whose families lived an hour or more away from the vaccination site had 0.50 times the odds of full vaccination status compared to those whose families lived 30–59 min away (95% CI: 0.28, 0.89). Although not statistically significant, two other factors had substantial associations with full vaccination status. Mothers who ever hesitated getting a vaccine for their child had children with decreased odds of full vaccination status (OR = 0.64, 95% CI: 0.39, 1.06), and mothers who did not think that vaccines were used to prevent disease had decreased odds of fully vaccinated children (OR = 0.51, 95% CI: 0.25, 1.04).

The 39% of women whose child was not fully vaccinated were asked why their child was not fully vaccinated (Table 4). Responses fell generally into three broad categories: lack of information, lack of motivation, and obstacles to vaccination. For example, fear of side reactions (36%) was the most common reason under lack of information; hearing rumors about vaccinations (28%) was the most common reason categorized as lack of motivation; and being too busy (31%) was the most common obstacle listed. Few described the vaccine site being too far (3%), place/time being unknown (3%), or there being a long waiting time (6%). Some reasons pertained to vaccine clinic issues, for example the vaccinators were absent (3%) or vaccines were not available (6%).

## 4. Discussion

In this study of children aged 12–23 months living in rural Worabe, the Southern Nation Nationality region of Ethiopia, we found well over half of children were fully vaccinated with the EPI recommended vaccines. This level of coverage is substantially higher than a similar study conducted in Wonago, in southern Ethiopia in 2008, where only 42% of children were vaccinated [10], or Ambo, in Oromia region in 2011, where 36% of children were fully vaccinated [11], but lower than the 76.0% full vaccination status found in the North Gondar Zone of Ethiopia in 2014 (76.0%) [20].

These differences in full vaccination highlight the gradient of immunization system performance across Ethiopia, given diverse religious, ethnolinguistic, and socioeconomic populations who have differential access to preventive care services and who have varying perceptions of the necessity of vaccination. Accordingly, Ethiopia faces unique difficulties in its efforts to reduce the burden of vaccine-preventable disease while also addressing short-term goals, such as 90% of districts in the country uniformly attaining at least 89% pentavalent Dose 3 coverage by 2018 [7].

Mothers indicated a variety of reasons for not completing the recommended vaccination schedule for their children. Some of these included attitudes about vaccines or misperceptions about vaccinations, such as fear of side reactions, hearing rumors, or being unaware of the need for vaccination or the need to return to the vaccine clinic to complete vaccine series. Other studies from Ethiopia have reported similar findings [19,20], which could, at least partially, be addressed by providing regular and integrated education about vaccines for mothers using both trained health professionals and others in allied health fields, such as traditional birth attendants or female Health Extension Workers. These community health workers have become an important source of health care information in Ethiopia, and have been a model for other Sub-Saharan African countries seeking to improve access to health care among under-served populations [21].

In terms of knowledge about vaccines, most mothers knew vaccination prevents disease, but relatively few were aware of other more specific facts such as when vaccinations are initiated or how many office visits are required. These findings are consistent with the study in Ambo, Ethiopia, in which more than half of mothers knew the purpose of immunization but far fewer knew the age for initiating childhood immunization (i.e., birth) [10]. This information has been shown to be relevant in another study from North Gondar Zone in Ethiopia which found full immunization status of children was higher among mothers who know the age at which the child become fully immunized than who did not know had greater awareness of completing their child’s vaccine series [20]. In general, mothers more informed about different aspects of vaccination are more likely to have their child fully vaccinated.

The strongest predictor for full vaccination coverage of children was the number of ANC visits. This finding is consistent with a number of other studies from Ethiopia, India, and Bangladesh, which have found that a greater number of ANC visits is associated with greater likelihood of having a child vaccinated [13,14,19]. These findings are likely reflective, in part, of underlying socioeconomic characteristics: wealthier individuals being more likely to access prenatal care and postnatal care, which includes vaccinations. However, in our study, we controlled for socioeconomic status in the multivariable regression, including potential confounders such as education, income, occupation, and farm land as a measure of wealth. Therefore, we have high confidence in our results in the context of the relationship between ANC visits and vaccination status. ANC visits represent an opportunity for mothers to receive information about vaccines, specifically, and their child’s future health, in general.

Mother’s occupation and ownership of farm land were also significantly linked to child’s full vaccination status. Farm land, a measure of wealth, could be a better indicator of socioeconomic status, than other variables we included, like education or income. House wives could have been more likely to have fully vaccinated children because they have more flexibility in their schedule to attend vaccination clinics or may have had more potential contact with Health Extension Workers. Mothers with a full-time occupation outside of the household may face more barriers, for example “lack of time”, which may prevent them from bringing their child to a vaccine clinic. Employers (with encouragement from the government) should recognize the importance of insuring families have time to take their children to vaccine clinics. Additionally, these clinics could maintain flexible hours of operation (including being open some nights or weekends) to help improve access for working families.

Other socioeconomic variables considered in this study were not significantly associated with full vaccination. Based on other studies, the relationship between socioeconomic variables and vaccination status in Ethiopia has been mixed. One other study found that few sociodemographic variables were related to vaccination [10], but others—including a study from North Gondar Zone and a study done in seven selected zone of Ethiopia—found that wealth, parity, maternal education, mothers’ knowledge of sessions needed to complete child vaccination, and sex of the child all have a significant association with various vaccination measures [19,20]. In a study of 45 GAVI-supported countries (a majority of which were in Africa), greater wealth (as measured by a wealth index) and greater education (for mothers and fathers) were associated with significantly higher coverage of DTP3 and measles-containing vaccine [17]. Therefore, it is likely that any program to improve vaccination coverage in Ethiopia will require targeting groups with low socioeconomic status.

### Limitations and Strengths

This study has certain limitations. Maternal recall may under- or overestimate immunization status of the child although it is unclear in which direction it would be for this study: the mother may forget the total number of doses the child received, or they may overstate the number because of social desirability bias. This study did not specifically look at problems from the health facility perspective, and did not include in-depth open-ended questions for participants to qualitative explain their problems with vaccination. However, we did specifically ask about vaccinations from several different angles—from ascertaining information sources to asking follow-up questions to mothers who had incompletely vaccinated children. For these reasons, we have a comprehensive picture of how a child comes to be incompletely vaccinated.

## 5. Conclusions

Full immunization of children aged 12–23 months in a Muslim-majority town in Ethiopia was below the national goals. Mothers indicate several reasons for not having their child immunized, many of which could be addressed, at least in part, with regular educational outreach in a variety of venues (hospital, clinics, and at home) and by a variety of health workers (doctors, nurses, and community health workers). Cross-promotion of vaccination and ANC could promote a healthier and more vaccinated childhood population.

## Figures and Tables

**Table 1:** Characteristics of children aged 12–23 months and their mothers, Worabe, Southern Nations, Nationalities, and Peoples’ Region, Ethiopia, 2016.

Characteristic	Count	%	Not Fully Immunized		Fully Immunized	
			Count	%	Count	%
Overall	484	100%	187	39%	297	61%
Sex of the child						
Male	243	50%	95	39%	148	61%
Female	241	50%	92	38%	149	62%
Child’s birth order						
First	149	31%	49	33%	100	67%
Second	104	21%	41	39%	63	61%
Third	81	17%	31	38%	50	62%
Fourth or more	150	31%	66	44%	84	56%
Mother has younger children						
Yes	13	3%	8	62%	5	38%
No	471	97%	179	38%	292	62%
Mother has older children						
No older child	154	32%	51	33%	103	67%
Yes, 3 years of age	124	26%	46	37%	78	63%
Yes, 4 years of age	105	22%	47	45%	58	55%
Yes, ≥5 years of age	101	21%	43	43%	58	57%
Parity						
1	147	30%	48	33%	99	67%
2	101	21%	41	41%	60	59%
3	73	15%	28	38%	45	62%
≥4	163	34%	70	43%	93	57%
Mothers age category						
Age 18–20 years	33	7%	13	39%	20	61%
Age 21–25 years	140	29%	54	39%	86	61%
Age 26–30 years	181	37%	70	39%	111	61%
Age ≥31 years	81	17%	27	33%	54	67%
I don’t know	49	10%	23	47%	26	53%
Marital status						
Married	474	98%	183	39%	291	61%
Not married	10	2%	4	40%	6	60%
Maternal Education						
No	176	36%	72	41%	104	59%
Elementary School	184	38%	72	39%	112	61%
High School	65	13%	20	31%	45	69%
College or more	59	12%	23	39%	36	61%
Religion						
Muslim	430	89%	171	40%	279	65%
Protestant	17	4%	5	29%	12	71%
Orthodox	35	7%	10	29%	25	71%
Monthly income						
≤1000 Birr	132	27%	58	44%	74	56%
1001–2000 Birr	106	22%	39	37%	67	63%
≥2001 Birr	123	25%	46	37%	77	63%
Didn’t reply	123	25%	44	36%	79	64%
Occupation						
Public or private employee	94	19%	41	44%	53	56%
Merchant	71	15%	35	49%	36	51%
House wife	245	51%	90	37%	155	63%
Students	7	1%	2	29%	5	71%
Farmer	39	8%	10	26%	29	74%
Day laborer	28	6%	9	32%	19	68%
Farm Land						
No	322	67%	138	43%	184	57%
Yes	162	33%	49	30%	113	70%
0.25 hectares	27	6%	11	41%	16	59%
0.5 hectares	40	8%	18	45%	22	55%
1 hectares	53	11%	12	23%	41	77%
1.5 hectares	3	1%	1	33%	2	67%
2 hectares	8	2%	2	25%	6	75%
Do not know size	27	6%	5	19%	22	81%
Number of antenatal care visits						
0	26	5%	22	85%	4	15%
1	104	21%	71	68%	33	32%
2	60	12%	24	40%	36	60%
3	86	18%	22	26%	64	74%
≥4	208	43%	48	23%	160	77%
Distance to vaccination site						
<30 min	157	32%	64	41%	93	59%
30–59 min	181	37%	59	33%	122	67%
≥60 min	146	30%	64	44%	82	56%

**Table 2:** Mothers’ awareness of vaccination and health care programs in Worabe, Southern Nations, Nationalities, and Peoples’ Region, Ethiopia, 2016.

Question	Count	%
Do you take your child to a health facility for regular check-ups?		
No	27	6%
Yes	457	94%
Where do you take your child when he/she is sick?		
Health facility	447	92%
Traditional Medicine	184	38%
Church/Mosque	99	20%
Holy water	27	6%
What have you heard about vaccines?		
Vaccination campaigns	246	51%
Vaccine benefits	390	81%
Place of vaccine	297	61%
Age for vaccine administration	203	42%
Vaccine schedule	190	39%
New vaccine information	65	13%
Source of information		
Newspaper	29	6%
Flyer	50	10%
Radio	93	19%
Community Leader	120	25%
TV	127	26%
Health Extension Worker	294	61%
Have you ever hesitated getting a vaccination for your child?		
No	224	46%
Yes	260	54%
At what age does child immunization begin?		
Incorrect answer	402	83%
Correct answer	82	17%
How many vaccinations sessions are needed for your child?		
Incorrect answer	85	18%
Correct answer	381	79%
I don’t know	18	4%
Vaccination is used to prevent disease		
No	71	15%
Yes	358	74%
I don’t know	55	11%

**Table 3:** Mother and child socioeconomic characteristics, maternal vaccine knowledge, and their relationship to full childhood immunization according to a multivariable logistic regression model, Worabe, Southern Nations, Nationalities, and Peoples’ Region, Ethiopia, 2016.

Characteristic	Adjusted Odds Ratio (95% Confidence Interval)	*p*-Value
Sex of the child		0.3658
Male	ref	
Female	1.22 (0.79, 1.90)	
Child’s birth order		0.2625
First	ref	
Second	0.72 (0.39, 1.35)	
Third	0.80 (0.38, 1.66)	
Fourth or more	0.50 (0.24, 1.02)	
Mother’s age		0.5204
Age 18–20 years	0.40 (0.13, 1.23)	
Age 21–25 years	0.56 (0.26, 1.18)	
Age 26–30 years	0.67 (0.34, 1.31)	
Age ≥31 years	0.40 (0.13, 1.23)	
I don’t know	0.65 (0.26, 1.61)	
Maternal education		0.4685
None	1.58 (0.66, 3.76)	
Elementary School	1.13 (0.53, 2.41)	
High School	1.70 (0.69, 4.15)	
College or more	ref	
Religion		0.2362
Muslim	ref	
Christian	1.55 (0.75, 3.21)	
Household monthly income		0.5777
≤1000 Birr	0.85 (0.45, 1.61)	
1001–2000 Birr	1.32 (0.68, 2.56)	
≥2001 Birr	ref	
Didn’t reply	1.13 (0.58, 2.20)	
Occupation		0.0325
House wife	ref	
Other	0.58 (0.35, 0.96)	
Farm Land		0.0008
No	ref	
Yes	2.51 (1.46, 4.30)	
Number of antenatal care visits		<0.0001
0	0.09 (0.03, 0.32)	
1	0.15 (0.08, 0.29)	
2	0.46 (0.23, 0.92)	
3	0.89 (0.48, 1.67)	
≥4	ref	
Distance to vaccination site		0.0440
<30 min	0.63 (0.37, 1.07)	
30–59 min	ref	
≥60 min	0.50 (0.28, 0.89)	
Do you take your child to a health facility for regular check-ups?		0.1185
No	0.49 (0.20, 1.20)	
Yes	ref	
Have you ever hesitated getting a vaccination for your child?		0.0831
No	ref	
Yes	0.64 (0.39, 1.06)	
Vaccination is used to prevent disease		0.0704
No	0.51 (0.25, 1.04)	
Yes	ref	
I don’t know	1.38 (0.67, 2.86)	

**Table 4:** Mothers’ reasons for not vaccinating child among those missing a child’s vaccination dose in Worabe, Southern Nations, Nationalities, and Peoples’ Region, Ethiopia, 2016.

Reason	Count	Proportion
Lack of information		
Unaware of need for vaccination	18	10%
Unaware of need to return for subsequent dose	20	11%
Place and/or time of vaccination unknown	6	3%
Fear of side reactions	67	36%
Wrong ideas about contra-indications	15	8%
Lack of motivation		
Postponed until another time	44	24%
No faith in vaccination	44	24%
Rumors	53	28%
Obstacles		
Place of vaccination too far	6	3%
Time of vaccination inconvenient	18	10%
Vaccinators absent	5	3%
Vaccine not available	11	6%
Mother too busy	58	31%
Family problem, including illness of the mother	14	7%
Child ill—not brought	12	6%
Long waiting time	12	6%

Note: Mothers could select multiple answers.
